# Stat3 Tyrosine 705 and Serine 727 Phosphorylation Associate With Clinicopathological Characteristics and Distinct Tumor Cell Phenotypes in Triple-Negative Breast Cancer

**DOI:** 10.3389/pore.2022.1610592

**Published:** 2022-08-09

**Authors:** Michaela Stenckova, Rudolf Nenutil, Borivoj Vojtesek, Philip J. Coates

**Affiliations:** ^1^ Masaryk Memorial Cancer Institute, Research Center for Applied Molecular Oncology (RECAMO), Brno, Czechia; ^2^ Department of Experimental Biology, Faculty of Science, Masaryk University, Brno, Czechia

**Keywords:** clinicopathological characteristics, triple-negative breast cancer, Stat3 tyrosine phosphorylation, Stat3 serine phosphorylation, tumor cell phenotypes

## Abstract

Signal transducer and activator of transcription 3 (Stat3) is responsible for many aspects of normal development and contributes to the development and progression of cancer through regulating epithelial cell identity and cancer stem cells. In breast cancer, Stat3 is associated with triple-negative breast cancers (TNBC) and its function has been related to the activation of p63, itself a marker of basal-like TNBC and a master regulator of stem cell activities. Stat3 activation is controlled by dual phosphorylation at tyrosine 705 (pTyr705) and serine 727 (pSer727), although it is unclear whether these have equivalent effects, and whether they are related or independent events. To address these issues, we investigated Stat3 phosphorylation at the two sites by immunohistochemistry in 173 patients with TNBC. Stat3 phosphorylation was assessed by automated quantitative measurements of digitized scanned images and classified into four categories based on histoscore. The results were analyzed for associations with multiple markers of tumor phenotype, proliferation, *BRCA* status, and clinicopathological characteristics. We show that the levels of pTyr705- and pSer727-Stat3 were independent in 34% of tumors. High pTyr705-Stat3 levels were associated with the luminal differentiation markers ERβ/AR and MUC1, whereas tumors with high levels of pSer727-Stat3 were more likely to be positive for the basal marker CK5/6, but were independent of p63 and were EGFR negative. Combined high pSer727- and low Tyr705-Stat3 phosphorylation associated with basal-like cancer. Although high Stat3 phosphorylation levels were associated with less aggressive tumor characteristics, they did not associate with improved survival, indicating that Stat3 phosphorylation is an unfavorable indicator for tumors with an otherwise good prognosis according to clinicopathological characteristics. These findings also show that pTyr705-Stat3 and pSer727-Stat3 associate with specific breast tumor phenotypes, implying that they exert distinct functional activities in breast cancer.

## Introduction

Stat3 is a member of the signal transducer and activator of transcription (Stat) family of transcription factors and exerts different and sometimes contrasting functions in normal and transformed cells (1–3). In the breast, Stat3 regulates mammary gland development, is activated during the proliferative phase of pregnancy, and plays roles during lactation and involution (3, 4). Stat3 activation is a transient and tightly regulated process in normal tissues, but occurs constitutively in many human tumors, including breast cancers, most notably in triple-negative breast cancer (TNBC). Stat3 signaling contributes to tumor cell survival, proliferation, migration, invasion, and chemoresistance, as well as influencing angiogenesis, immunosuppression, and cancer stem cell (CSC) self-renewal/differentiation (1, 5, 6). Whilst Stat3 regulates many genes and processes, its regulation of normal and cancer stem cells has been linked particularly with direct transcriptional activation of ΔNp63, itself a regulator of normal and CSCs and associated specifically with basal-like TNBC (1, 7–10).

Despite the observations for oncogenic roles, immunohistochemical studies of Stat3 give conflicting results for prognosis (11–15). Importantly, there is increasing preclinical and clinical evidence that Stat3 inhibitors reduce breast cancer growth and metastasis (6,16), indicating the potential clinical value of measuring Stat3 activity. Stat3 activity is controlled by phosphorylation of tyrosine 705 (pTyr705-Stat3), which leads to dimerization, translocation to the nucleus, and transcriptional activation of target genes (17). Stat3 is also phosphorylated on serine 727 (pSer727-Stat3), and phosphorylation at both sites provides maximal activity (18, 19). Thus, it is generally thought that the two phosphorylation events act in concert. However, Ser727 phosphorylation can reduce Tyr705-Stat3 phosphorylation (20) and Stat3 can be activated through Ser727 phosphorylation in the absence of pTyr705 (21–23). Moreover, pSer727-Stat3 has been reported to localize to mitochondria and the endoplasmic reticulum to mediate their functions, including mitochondrial apoptosis pathways (24). Stat3 phosphorylation also has roles in defining cell identity and differentiation, where pTyr705-Stat3 induces an epithelial phenotype and pSer727-Stat3 a mesenchymal phenotype in pancreatic and lung cancers (25), and pTyr705-Stat3 maintains pluripotency whilst pSer727-Stat3 induces differentiation in embryonic stem cells (26).

These findings suggest that Tyr705- and Ser727-Stat3 phosphorylation may be independent regulators of cancer cell phenotypes. Thus, we studied pTyr705- and pSer727-Stat3 in a set of well-characterized TNBCs and correlated the relative levels of each Stat3 phosphorylation with each other and with clinicopathological and phenotypic features. We show that pTyr705-Stat3 and pSer727-Stat3 are often independent of each other and influence the basal/luminal phenotype of TNBC cells in opposite directions. We also show that high levels of pStat3 are associated with good prognostic indicators but not with improved survival suggesting it may be a useful target for therapeutic intervention in these patients.

## Materials and Methods

### Patient Tissues and Immunohistochemical Staining

Tissue microarrays (TMAs) were prepared from excess material from formalin-fixed and paraffin-embedded histological tissue blocks of TNBCs from patients before treatment, as described previously (27). The samples had been collected from unselected consecutive cases of TNBCs treated at Masaryk Memorial Cancer Institute (MMCI) between 2004 and 2009. TMAs contained two tissue cores of 1.5 mm in diameter for each cancer. In accordance with the Declaration of Helsinki, all patients had provided written informed consent to use their leftover material for research, and permission for the use of these excesses and redundant anonymized human tissue samples was approved by the MMCI ethical committee and the Biobank of clinical samples at MMCI (ethical approval for grant number NS/10357-3).

Sections were deparaffinized and rehydrated. Endogenous peroxidase was blocked in hydrogen peroxide (3%) and antigen retrieval was performed by boiling for 20 min in EDTA (1 mM, pH 8.0). Primary antibodies were applied overnight at 4°C and EnVision^+^ HRP reagents with DAB+ (Dako, Agilent Technologies, Santa Clara, CA, United States) were used for visualization. Primary antibodies to pTyr705-Stat3 (Cell Signaling Technology, Danvers, MA, United States; #9145, 1:200) or pSer727-Stat3 (Santa Cruz Biotechnology, Dallas, TX, United States; sc-8001-R, 1:500) were used. These antibodies have been independently characterized for specificity and performance in immunostaining previously (28–30). Slides were counterstained with hematoxylin, dehydrated and cleared in xylene, coverslipped with Entellan, and scanned by a Pannoramic Midi Slide Scanner (3D Histech, Budapest, Hungary). Patient details and clinicopathological characteristics were described previously (27).

### Quantitative Analysis of Stat3 Phosphorylation and Statistical Evaluation

QuPath (0.1.2) was used for quantification (https://qupath.github.io/) (31). TMAs were manually annotated for cancer cells and representative populations were selected from each of the two tissue cores for each patient. Positive cells were detected using the parameters presented in Supplementary Table S1 and the histoscore (H-score) was calculated for nuclear signal localization. The weighted average H-score was calculated from the replicate tissue cores for each tumor. Data were normalized using the average H-score value/standard deviation for each antibody separately to take into account differences in antibody sensitivities and staining characteristics. Thus, a normalized value of zero represents the average H-score for that antibody, with values below zero representing tumors with lower than average staining and above zero representing above-average values. Above and below-average scores were subdivided into two groups each according to the following cut-offs: group 1 < −0.9; group 2 > -0.9 < 0; group 3 > 0 < 0.9; group 4 > 0.9.

Associations between pTyr705- and pSer727-Stat3 and clinicopathological data were evaluated using chi-square tests or Student´s t-tests in the indicated cases. Kaplan-Meier and Mantel-Cox log-rank tests were used for survival data (32). We initially evaluated different cut-offs derived from normalized H-scores for comparison of pStat3 histoscores with other immunohistochemical markers and with clinicopathological variables. Unless indicated otherwise, results provide values for high versus low dichotomization (low = group 1 and 2 combined; high = group 3 and 4). For survival analyses and clinical features such as clinical stage, grade, node status, tumor size and relapse, very high was used as the cut-off (low = groups 1–3; high = group 4). For cytokeratins (CK) CK5/6, CK8/18, and CK14, very low was used as the cut-off (low = group 1; high = groups 2–4).

## Results

Immunostaining data were obtained for pTyr705- and/or pSer727-Stat3 in 173 TNBCs. Data were available for both in 151 tumors. Uninterpretable spots were due to loss of tissue on the array or insufficient numbers of tumor cells.

pTyr705-Stat3 localized to the nuclei of tumor cells and stromal cells including lymphocytes and fibroblasts, which served as convenient internal controls for staining. pTyr705-Stat3 was not seen in the cytoplasm. In contrast, pSer727-Stat3 was observed in the nucleus and the cytoplasm of tumor cells and stromal cells, compatible with previous data on the location of these two phosphorylated forms of Stat3 (25). To investigate the effects of phosphorylation events specifically on transcriptional activity within tumor cells, only nuclear labeling of tumor cells was used for quantification of the selected areas in QuPath, resulting in an H-score for each phosphorylation event for each tumor. H-scores for pTyr705-Stat3 ranged from 1.59 to 213.20 (mean 43.59 ± 36.02 SD) and from 8.10 to 242.75 for pSer727-Stat3 (mean 120.50 ± 53.20 SD). For comparison with clinicopathological variables, nuclear H-scores were used to divide tumors into groups above and below the mean value for each phosphorylation.

**FIGURE 1:**
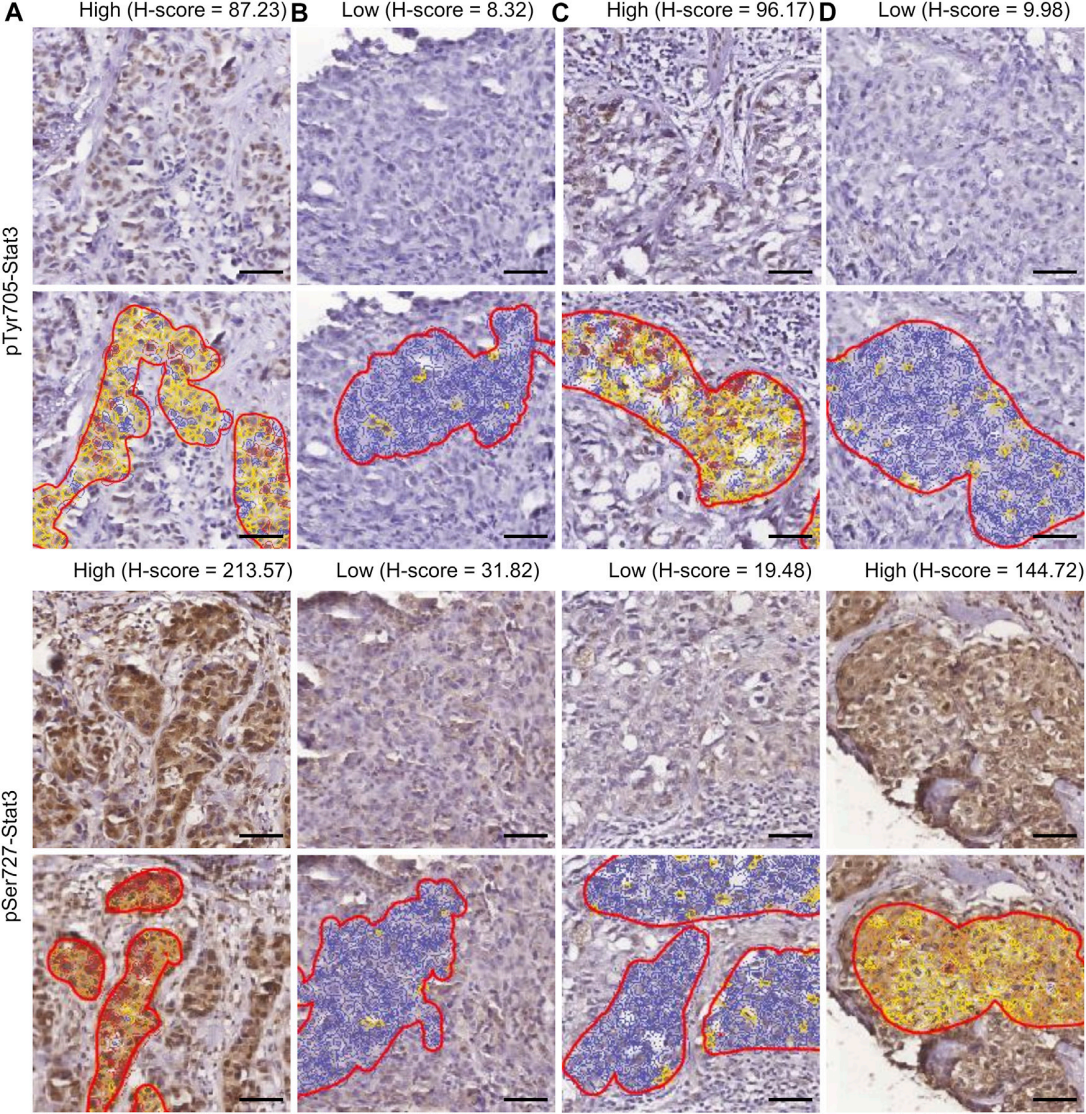
Immunohistochemistry of Stat3 phosphorylation in TNBC. The four patterns of staining and corresponding examples of QuPath selections are shown. Selected areas are marked by manually drawn red lines. Cells are color-coded according to the intensity of DAB staining for calculation of the H-score within QuPath. **(A)** High pTyr705-/high pSer727-Stat3; **(B)** low pTyr705-/low pSer727-Stat3; **(C)** high pTyr705-/low pSer727-Stat3; **(D)** low pTyr705-/high pSer727-Stat3. Scale bar = 50 μm.

**FIGURE 2:**
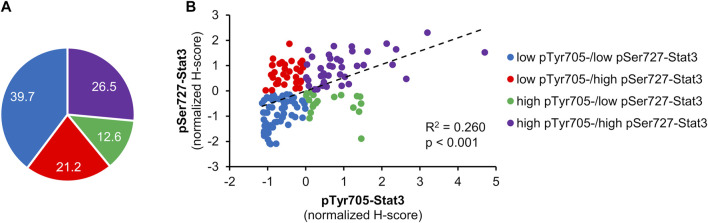
Correlation between pTyr705- and pSer727-Stat3. Normalized H-scores were calculated for each tumor, whereby a value of zero is the average score for each antibody across the patient cohort. Samples are color-coded; blue shows tumors that according to their normalized H-scores belong to the low pTyr705-/low pSer727-Stat3 group; purple shows tumors within the high pTyr705-/high pSer727-Stat3 group; red shows low pTyr705-/high pSer727-Stat3 group; green shows tumor samples belonging to the high pTyr705-/low pSer727-Stat3 group. **(A)** The representation of individual groups. **(B)** Normalized H-score values and correlation of pTyr705- and pSer727-Stat3. The dotted line shows the association trendline.

Figure 1 displays examples of pTyr705- and pSer727-Stat3 immunohistochemical staining and corresponding examples of QuPath selections. We initially examined whether the two phosphorylation events were concordant or were independent of each other. A common trend of staining was seen in 100 cases (66%), in which low pTyr705- and low pSer727-Stat3 were present in 60 tumors, and high pTyr705- and high pSer727-Stat3 in 40 samples. An opposite trend was observed in 51 patients (34%), with low pTyr705- and high pSer727-Stat3 in 32, and high pTyr705- and low pSer727-Stat3 staining intensity in 19 tumors (Figure 2A). Pearson correlation coefficient showed a positive correlation between pTyr705- and pSer727-Stat3 normalized histoscores within individual tumors (r = 0.509; Figure 2B). Whilst the correlation is statistically significant, it is also evident that many individual tumors (over one-third of the cohort) showed discordant values for phosphorylation at the two sites.

### Stat3 Shows Phosphorylation-Specific Clinicopathological Associations

Data were available for pTyr705-Stat3 in 4 lobular-pleiomorphic, 3 metaplastic, 3 micropapillary, 3 papillary, 5 pleiomorphic tumors, 33 tumors with medullary features, and 117 tumors of no-special type. Data for pSer727-Stat3 were available in 3 lobular-pleiomorphic, 5 metaplastic, 2 micropapillary, 3 papillary, 6 pleiomorphic tumors, 35 tumors with medullary features, and 111 no-special type cancers. There were no significant associations between pTyr705- or pSer727-Stat3 levels and histological subtype, although the numbers of each special histological subtype were very low (Supplementary Table S2).

**FIGURE 3:**
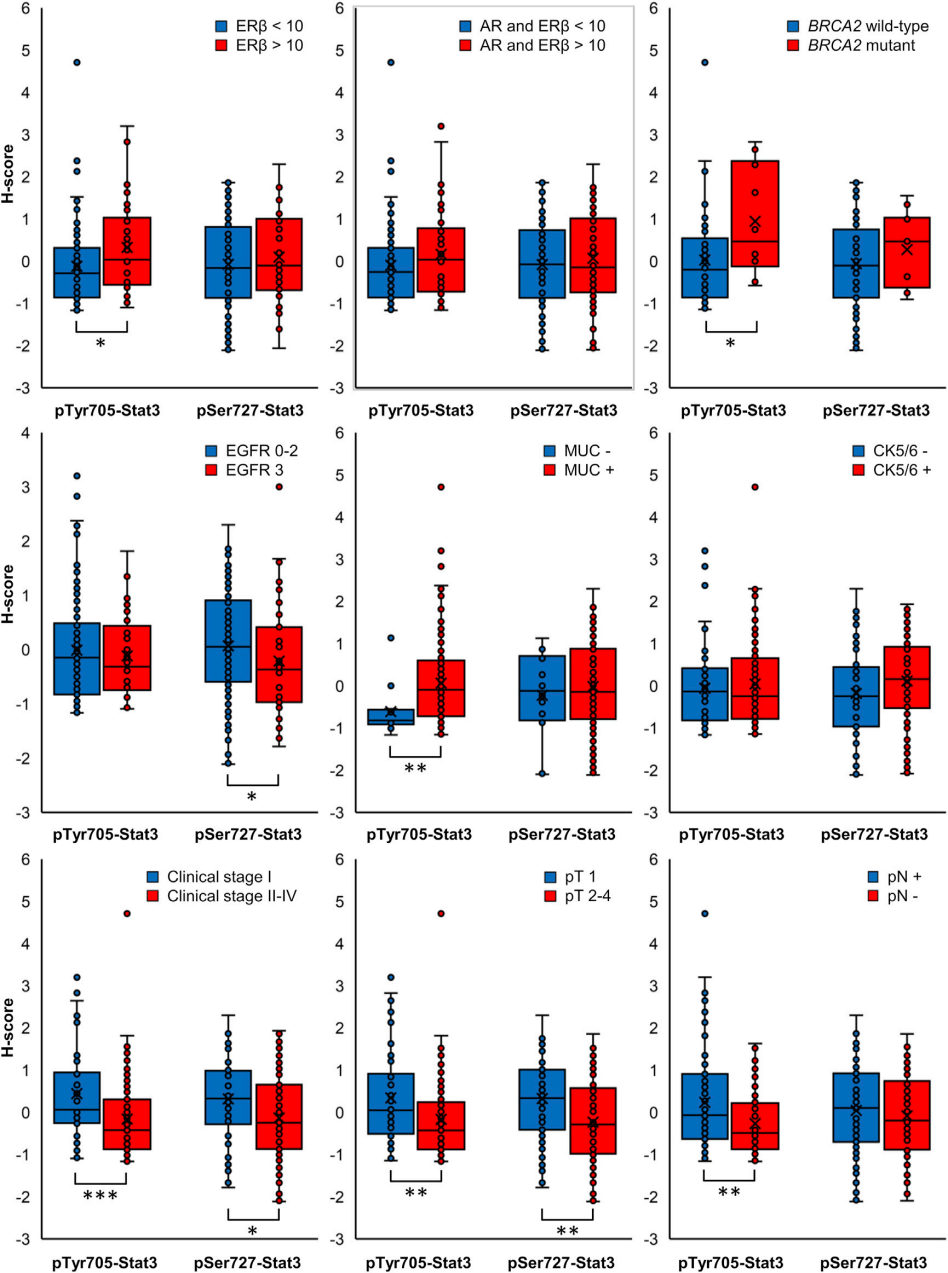
Box plots displaying the distribution of ungrouped normalized H-scores. Clinicopathological characteristics in association with pTyr705- or pSer727-Stat3 with *p*-value < 0.05 for at least one phosphorylated Stat3 form are included (*p < 0.05, **p < 0.01, ***p < 0.001).

The clinicopathological characteristics in association with individual pTyr705- and pSer727-Stat3 groupings are shown in Table 1 and Supplementary Table S3. Figure 3 shows data for the clinicopathological characteristic that were significantly associated with either pTyr705- or pSer727-Stat3 levels (*p* < 0.05). Clinicopathological correlations with the combined levels of both pTyr705- and pSer727-Stat3 are shown in Table 2 and Supplementary Table S4.

**TABLE 1:** Clinicopathological characteristics in association with either pTyr705- or pSer727-Stat3 levels.

	pTyr705-Stat3			pSer727-Stat3		
	Low	High	*p*-value	Low	High	*p*-value
AR > 10	14	13	0.585	16	11	0.430
AR < 10	68	50		60	58	
ERβ > 10	14	20	**0.029**	20	16	0.700
ERβ < 10	68	41		56	52	
AR and ERβ > 10	25	31	**0.022**	31	26	0.652
AR and ERβ < 10	57	32		45	44	
BLBC +	76	54	0.093	67	62	0.896
BLBC −	4	8		7	6	
*BRCA1* mutant	18	20	0.300	16	19	0.318
*BRCA1* wild-type	27	19		24	18	
*BRCA2* mutant	2	7	**0.039**	5	5	0.917
*BRCA2* wild-type	36	25		29	27	
Clinical stage I	32	16	**0.002**	32	13	0.267
Clinical stage II-IV	100	14		92	24	
Grade 1–2	10	3	0.659	9	5	0.236
Grade 3	122	27		115	32	
EGFR 0-2	75	56	0.678	63	69	**0.038**
EGFR 3	22	14		23	11	
MUC1 +	74	63	**0.012**	72	64	0.918
MUC1 −	11	1		6	5	
p53 mutant	58	41	0.555	57	44	0.142
p53 wild-type	24	21		19	25	
Relapse yes	35	6	0.434	34	12	0.572
Relapse no	95	24		89	25	
ΔNp63 +	8	9	0.325	9	7	0.724
ΔNp63 −	91	62		80	75	
TAp63 +	7	8	0.326	8	7	0.938
TAp63 −	92	62		80	73	
CK5/6 +	14	82	0.484	14	82	**0.036**
CK5/6 −	12	52		18	46	
CK8/18 +	21	121	0.088	32	109	0.880
CK8/18 −	3	5		2	6	
CK14 +	14	63	0.674	15	60	0.861
CK14 −	14	75		19	71	
pN +	56	5	**0.006**	51	14	0.537
pN −	62	22		60	21	
pT 1	39	15	**0.029**	36	19	**0.016**
pT 2-4	79	12		77	16	

BLBC, basal-like breast cancer; pN, pathological regional lymph-nodes; pT, pathological primary tumor size. Bold indicates statistical significance (*p* < 0.05).

**TABLE 2:** Clinicopathological characteristics in association with combined pTyr705- and pSer727-Stat3.

	Low pTyr705-/low pSer727-Stat3	High pTyr705-/high pSer727-Stat3	*p*-value	Low pTyr705-/high pSer727-Stat3	High pTyr705-/low pSer727-Stat3	*p*-value
AR > 10	11	10	0.553	1	3	0.110
AR < 10	40	27		25	13	
ERβ > 10	10	10	0.334	4	6	0.102
ERβ < 10	41	25		22	10	
AR and ERβ > 10	18	19	0.132	5	8	**0.036**
AR and ERβ < 10	33	18		21	8	
BLBC +	47	33	0.675	24	12	**0.045**
BLBC −	3	3		1	4	
*BRCA1* mutant	10	10	0.372	8	6	0.925
*BRCA1* wild-type	14	8		10	7	
*BRCA2* mutant	1	4	0.074	1	2	0.364
*BRCA2* wild-type	20	12		14	9	
CK5/6 +	7	66	0.444	6	4	0.124
CK5/6 −	6	36		4	10	
CK8/18 +	13	94	0.137	5	14	0.117
CK8/18 −	2	4		1	0	
CK14 +	8	49	0.606	4	5	0.831
CK14 −	7	57		6	9	
Clinical stage I	24	8	**0.002**	4	4	0.399
Clinical stage II-IV	75	4		16	8	
EGFR 0-2	40	31	0.135	28	15	0.472
EGFR 3	19	7		3	3	
Grade 1–2	8	3	0.064	2	0	0.258
Grade 3	91	9		18	12	
MUC1 +	46	35	0.135	23	17	0.096
MUC1 −	6	1		4	0	
p53 mutant	39	21	0.072	16	13	0.180
p53 wild-type	12	15		10	3	
Relapse yes	27	1	0.149	8	3	0.387
Relapse no	71	11		12	9	
TAp63 +	5	4	0.714	1	0	N/A
TAp63 −	55	34		31	18	
ΔNp63 +	6	4	0.933	2	1	0.885
ΔNp63 −	54	34		30	18	
pN +	44	3	0.155	8	2	0.168
pN −	44	8		12	10	
pT 1	26	8	**0.004**	7	4	0.923
pT 2-4	63	3		13	8	

BLBC, basal-like breast cancer; pN, pathological regional lymph-nodes; pT, pathological primary tumor size. Bold indicates statistical significance (*p* < 0.05).

### pTyr705-Stat3

High levels of pTyr705-Stat3 were associated with ERβ positivity (*p* = 0.029) and with combined ERβ and androgen receptor (AR) positivity (*p* = 0.022). Interestingly, dual ERβ and AR positivity correlated specifically with high levels of pTyr705- and low levels of pSer727-Stat3 (*p* = 0.036). Furthermore, tumors with high levels of pTyr705-Stat3 were more likely to be mucin-1 (MUC1, CA 15-3) positive (*p* = 0.012). The proportion of patients carrying *BRCA2* mutation was higher in the high pTyr705-Stat3 tumor group (*p* = 0.039). High pTyr705-Stat3 also correlated with less aggressive tumor characteristics such as smaller tumor size (*p* = 0.029), lower clinical stage (*p* = 0.002), and absence of lymph node metastases (*p* = 0.006).

### pSer727-Stat3

Tumors with high levels of pSer727-Stat3 were more likely to be positive for CK5/6 (*p* = 0.036) but negative for EGFR (*p* = 0.038). High pSer727-Stat3 was associated with smaller tumor size (*p* = 0.016) and lower clinical stage when high levels of pTyr705-Stat3 were also present (*p* = 0.002), but pSer727-Stat3 did not associate with lymph node metastasis. Patients with basal-like cancers showed high levels of pSer727- and low levels of pTyr705-Stat3 (*p* = 0.045).

**FIGURE 4:**
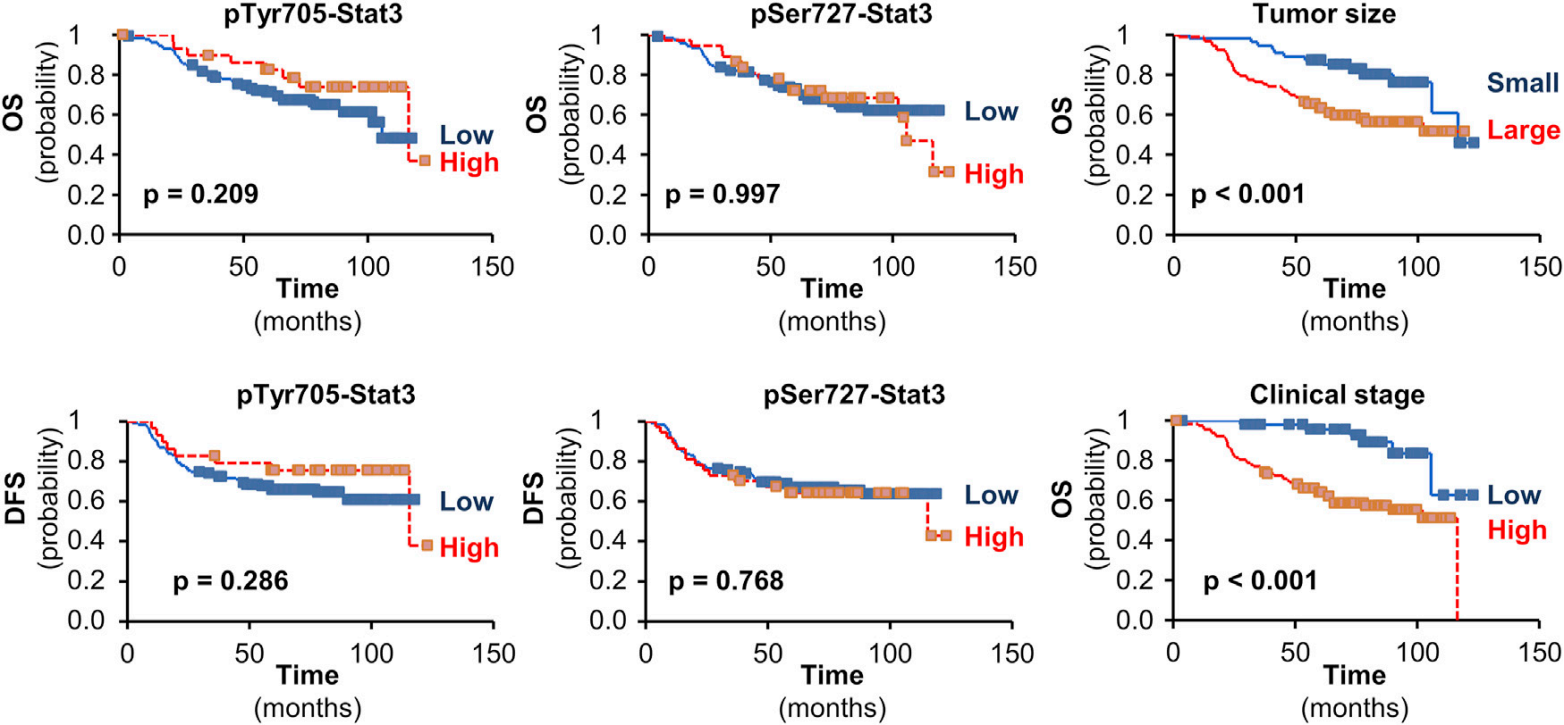
The impact of pStat3 expression on survival. Kaplan-Meier plots for overall (OS) or disease-free survival (DFS) in TNBCs according to pTyr705- or pSer727-Stat3. Kaplan-Meier plots are also shown for the impact of tumor size (pT 1 vs. pT 2-4) or clinical stage (I vs. II-IV) on OS in the set of patients available for pTyr705-Stat3 (similar results were obtained for the set of patients available for pSer727-Stat3). Mantel-Cox log-rank tests were used and *p*-values are shown.

Neither pTyr705- nor pSer727-Stat3 showed an association with TAp63 or ΔNp63 levels, or with p53 status. Furthermore, although Stat3 phosphorylation of Tyr705 and Ser727 was associated with less aggressive tumor characteristics such as tumor size or clinical stage, neither phosphorylation showed an association with survival (Figure 4).

## Discussion

The role of Stat3 in cancer is controversial, with several studies demonstrating an oncogenic role in the development of various cancers, whereas others have indicated that Stat3 behaves as a tumor suppressor (1, 25). These contrasting data imply dependency on tumor type, stage of the disease, and local microenvironment; Stat3 activation or inactivation can each support disease progression depending on such factors (33, 34). In breast cancer, Stat3 is more often overactive in TNBCs than other types (5) and has been implicated in inducing CSC properties (35).

Stat3 activation involves phosphorylation to allow dimerization, nuclear transport and binding to response elements in the promoters of target genes. In general, it is thought that Tyr705 phosphorylation is the initial event for Stat3 activation, followed by Ser727 phosphorylation to provide higher activity. Thus, most immunochemical studies evaluate total Stat3, or pTyr705-Stat3 as a general indicator of activity and only exceptionally pSer727-Stat3. However, data suggest that these two events have distinct functional effects during embryonic stem cell differentiation and in dictating epithelial cell identity in pancreatic and lung cancers (25, 26).

In our study, we found that pTyr705- and pSer727-Stat3 are related events that are often associated with each other. On the other hand, individual tumors show discrepancies, where one phosphorylation event occurs in the absence of the other, suggesting that different signaling events are operating in these tumors. Most importantly, each phosphorylation is associated with a distinct tumor cell phenotype, indicating that pTyr705- and pSer727-Stat3 regulate distinct pathways within the tumor. In particular, high levels of pTyr705- but not pSer727-Stat3 were associated with ERβ alone and with ERβ and AR combined. Our results further showed that patients with high levels of pTyr705-Stat3 were more likely to be MUC1-positive. High-level AR is the defining feature of luminal-AR TNBCs (36) and Burstein et al. (37) further characterized this phenotype by showing these tumors also over-express MUC1. Thus, pTyr705-Stat3 associates with an epithelial cell phenotype in TNBC, analogous to its specific effects in lung and pancreatic cancers (25). It is also important to note that transmembrane C-terminal MUC1 (MUC1-c) is necessary for Tyr705-Stat3 phosphorylation in breast cancer cells and promotes Stat3-mediated transcription in an auto-inductive regulatory loop (38). Thus, whether pTyr705-Stat3 is a cause or effect of this specific phenotype will require further study. We also found an association between high levels of pTyr705-Stat3 and *BRCA2* mutation, which is more common in ER-positive luminal B tumors (39). Moreover, consistent with our findings, pTyr705-Stat3 has been reported recently to disrupt CHK1 phosphorylation, thereby impairing BRCA2-mediated RAD51 recruitment for homologous recombination repair of double-strand DNA breaks (40).

In contrast to the association of pTyr705-Stat3 with an AR^+^/ERβ^+^/MUC1^+^ epithelial phenotype, pSer727-Stat3 is associated with a basal cell phenotype, again analogous to the mesenchymal phenotype induced by pSer727-Stat3 in other cancers (25). In particular, tumors with high levels of pSer727-Stat3 were more often CK5/6-positive, the most useful marker for the basal subgroup of TNBC (41). On the other hand, basal cancers, especially the BL2 subtype, are characterized by high levels of EGFR (42), whereas high levels of pSer727-Stat3 were associated with low EGFR in our samples. These findings are in agreement with the increasing recognition of intra-tumor heterogeneity that can be caused by a variety of cell-intrinsic and extrinsic traits that, together with inter-tumor heterogeneity, significantly affect a patient’s prognosis, therapy response and clinical outcome (43–45). In this respect, the use of a tissue core in a tissue microarray may not provide an overall picture of the entire tumor. In our study, this potential problem of intra-tumor heterogeneity was mitigated by using two independent cores taken from non-adjacent regions of the tumor for all patients, and this approach has been shown to accurately represent the overall tumor phenotype (46, 47).

Stat3 is an important regulator of CSC phenotypes (1, 48). It is becoming evident that various CSC subtypes exist in breast and other cancers, have different effects on prognosis, and are another source of tumor heterogeneity (43, 49–51). For example, ΔNp63 marks a specific subtype of basal/mesenchymal CSCs in breast cancer, as opposed to the luminal/epithelial CSC subtype in these tumors (27, 52, 53) and there is a similar population of ΔNp63-positive basal/mesenchymal CSCs in prostate adenocarcinomas (54). ΔNp63 and Stat3 are involved in several common pathways regulating CSC properties (1, 7), Stat3 is a direct regulator of ΔNp63 transcription (9, 10) and they cooperate in regulating epithelial cell identity in KRas-driven lung and pancreatic cancers (25), whilst ΔNp63 overexpression itself alters pTyr705- and Ser727-Stat3 levels in TNBC cells (55). However, our results show that pStat3 is not associated with ΔNp63 (or TAp63) in TNBC. Thus, although either pSer727-Stat3 or ΔNp63 may associate with basal cell phenotypes in TNBC they are independent of each other, suggesting at least two transcriptional activation routes to this phenotype and providing further evidence for heterogeneity of CSC phenotypes in breast cancer.

High pTyr705-Stat3 is associated with less aggressive tumor characteristics such as smaller tumor size, lower clinical stage, and absence of lymph node metastasis, similar to some previous studies (11, 56–58), but different from others (59). Levels of pSer727-Stat3 were associated with tumor size, and, together with high levels of pTyr705-Stat3 also with clinical stage. This is inconsistent with data from Yeh et al. (30), where high pSer727-Stat3 was correlated with larger tumors and higher clinical stages. Contributors to these differences include method sensitivity and the scoring systems and cut-offs used (58). In our study, we employed an objective rather than subjective approach to delineate pStat3 levels based on the mean values obtained for each antibody.

Our data show that although pStat3 phosphorylated at either site is associated with less aggressive tumor characteristics that are independently associated with better survival, such as tumor size or clinical stage, pStat3 itself is not associated with survival. Thus, these data imply that high pStat3 is an unfavorable indicator in tumors with a good prognosis according to their clinicopathological factors. This observation may relate to the ability of Stat3 to induce drug resistance (60, 61). Furthermore, recent data indicate that cancer cells dynamically exploit Stat3 activity, whereby both Stat3 activation and inactivation support cancer progression in a time- and space-dependent manner (34).

In conclusion, we show that pTyr705- and pSer727-Stat3 are associated with more favorable clinicopathological features but not with better survival, inferring its negative role in TNBC prognosis. Our results further indicate that Stat3 phosphorylation at Tyr705 and Ser727 have different effects on the phenotype of TNBCs, analogous to their distinct roles in embryonic stem cell self-renewal/lineage commitment (26) and cellular phenotype in pancreatic and lung cancers (25). In turn, these data indicate complex regulation and roles for Stat3 in TNBC, helping to explain the discordant results of studies that report the impacts of total Stat3 levels or single phosphorylation site analysis. The site-specificity of Stat3 phosphorylation should be taken into account in clinical trials that aim to disrupt Stat3 signaling in breast and other cancers (6, 48).

## Data Availability

The original contributions presented in the study are included in the article/Supplementary Material, further inquiries can be directed to the corresponding authors.
