# Supplementary material for: Stat3 Tyrosine 705 and Serine 727 Phosphorylation Associate With Clinicopathological Characteristics and Distinct Tumor Cell Phenotypes in Triple-Negative Breast Cancer
Source: Pathol Oncol Res. 2022 Aug 9;28:1610592. doi: 10.3389/pore.2022.1610592 (PMC9395589; doi:10.3389/pore.2022.1610592)
Supplement: Supplementary file 1 [file DataSheet1.docx]

**Supplementary Table S1** QuPath settings

|  | **pTyr705-Stat3** | **pSer727-Stat3** |
| --- | --- | --- |
| **Setup parameters** |  |  |
| Choose detection image | Optical density sum | Optical density sum |
| Requested pixel size | 0.5 µm | 0.5 µm |
| **Nucleus parameters** |  |  |
| Background radius | 8 µm | 8 µm |
| Median filter radius | 0 µm | 0 µm |
| Sigma | 1.5 um | 1.5 um |
| Minimum area | 10 µm^2^ | 10 µm^2^ |
| Maximum area | 400 µm^2^ | 400 µm^2^ |
| **Intensity parameters** |  |  |
| Threshold | 0.1 | 0.1 |
| Max background intensity | 2 | 2 |
| Split by shape | Yes | Yes |
| Exclude DAB (membrane staining) | No | No |
| **Cell parameters** |  |  |
| Cell expansion | 5 µm | 5 µm |
| Include cell nucleus | Yes | Yes |
| **General parameters** |  |  |
| Smooth boundaries | Yes | Yes |
| Make measurements | Yes | Yes |
| **Intensity threshold parameters** | |  |
| Score compartment | Nucleus: DAB OD mean | Nucleus: DAB OD mean |
| Threshold 1+ | 0.1 | 0.25 |
| Threshold 2+ | 0.2 | 0.4 |
| Threshold 3+ | 0.25 | 0.6 |
| Single threshold | No | No |

**Supplementary Table S2** pTyr705- and pSer727-Stat3 in association with histological subtypes

| **Histological subtype** | **pTyr705-Stat3** | | **pSer727-Stat3** | |
| --- | --- | --- | --- | --- |
|  | **low** | **high** | **low** | **high** |
| **LOBUL-PLEIO** | 1 (25.0%) | 3 (75.0%) | 1 (33.3%) | 2 (66.7%) |
| **MEDUL** | 19 (57.6%) | 14 (42.4%) | 19 (54.3%) | 16 (45.7%) |
| **METAPLASTIC** | 3 (100.0%) | 0 (0.0%) | 3 (60.0%) | 2 (40.0%) |
| **MICROP** | 1 (33.3%) | 2 (66.7%) | 2 (100.0%) | 0 (0.0%) |
| **NST** | 66 (56.4%) | 51 (43.6%) | 57 (51.4%) | 54 (48.6%) |
| **PAPIL** | 2 (66.7%) | 1 (33.3%) | 1 (33.3%) | 2 (66.7%) |
| **PLEIO** | 5 (100.0%) | 0 (0.0%) | 4 (66.7%) | 2 (33.3%) |

LOBUL-PLEIO, lobular-pleiomorphic; MEDUL, medullary features; MICROPAP, micropapillary; NST, no special type; PAPIL, papillary; PLEIO, pleiomorphic

**Supplementary Table S3** Additional clinicopathological characteristics in association with relative pTyr705- or pSer727-Stat3 levels

|  | **pTyr705-Stat3** | | | **pSer727-Stat3** | | |
| --- | --- | --- | --- | --- | --- | --- |
|  | **low** | **high** | **p-value** | **low** | **high** | **p-value** |
| **Age (average)** | 53.3 | 53.0 | **0.984*** | 54.1 | 53.0 | **0.438*** |
| **Age (median)** | 55.5 | 51.3 |  | 56.0 | 53.5 |  |
| **Bcl-2 +** | 34 | 20 | **0.290** | 29 | 26 | **0.938** |
| **Bcl-2 -** | 38 | 33 |  | 39 | 34 |  |
| **BLBC CK +** | 57 | 41 | **0.709** | 48 | 49 | **0.230** |
| **BLBC CK -** | 23 | 19 |  | 26 | 17 |  |
| **Cyclin D1 > 30** | 41 | 28 | **0.628** | 40 | 29 | **0.231** |
| **Cyclin D1 < 30** | 41 | 33 |  | 36 | 39 |  |
| **Ki67 (average)** | 62.3 | 62.5 | **0.993*** | 59.5 | 64.4 | **0.278*** |
| **Ki67 (median)** | 65.0 | 60.5 |  | 55.8 | 64.5 |  |
| **MDM2 > 5** | 58 | 43 | **0.858** | 53 | 53 | **0.265** |
| **MDM2 < 5** | 24 | 19 |  | 23 | 15 |  |
| **p16 1+2** | 15 | 11 | **0.932** | 15 | 13 | **0.891** |
| **p16 3** | 67 | 51 |  | 61 | 56 |  |
| **p27/Kip1 +** | 74 | 53 | **0.664** | 72 | 58 | **0.957** |
| **p27/Kip1 -** | 9 | 5 |  | 6 | 5 |  |
| **PD-L1 +** | 5 | 3 | **0.780** | 3 | 3 | **0.905** |
| **PD-L1 -** | 92 | 68 |  | 85 | 77 |  |
| **PTEN +** | 58 | 44 | **0.639** | 53 | 45 | **0.694** |
| **PTEN -** | 34 | 22 |  | 30 | 29 |  |
| **SMA 0+1** | 88 | 67 | **0.301** | 80 | 70 | **0.324** |
| **SMA 2+3** | 8 | 3 |  | 6 | 9 |  |
| **TILs +** | 35 | 24 | **0.577** | 28 | 27 | **0.731** |
| **TILs -** | 47 | 39 |  | 49 | 42 |  |
| **Vimentin +** | 54 | 36 | **0.486** | 45 | 45 | **0.423** |
| **Vimentin -** | 32 | 27 |  | 34 | 26 |  |

Age, age at diagnosis (years); BLBC CK, basal-like breast cancer (based on cytokeratins); TILs, tumor-infiltrating lymphocytes

* Student´s t-test

**Supplementary Table S4** Additional clinicopathological characteristics in association with combined pTyr705- and pSer727-Stat3 levels

|  | **low pTyr705-/ low pSer727-Stat3** | **high pTyr705-/ high pSer727-Stat3** | **p-value** | **low pTyr705-/ high pSer727-Stat3** | **high pTyr705-/ low pSer727-Stat3** | **p-value** |
| --- | --- | --- | --- | --- | --- | --- |
| **Age (average)** | 54.4 | 53.6 | **0.762** | 50.7 | 52.1 | **0.751** |
| **Age (median)** | 55.5 | 52.0 |  | 52.5 | 51.0 |  |
| **Bcl-2 +** | 18 | 10 | **0.529** | 13 | 7 | **0.412** |
| **Bcl-2 -** | 25 | 19 |  | 11 | 10 |  |
| **BLBC CK +** | 34 | 24 | **0.801** | 20 | 10 | **0.217** |
| **BLBC CK -** | 16 | 10 |  | 5 | 6 |  |
| **Cyclin D1 > 30** | 27 | 14 | **0.238** | 12 | 7 | **0.879** |
| **Cyclin D1 < 30** | 24 | 21 |  | 14 | 9 |  |
| **Ki67 (average)** | 60.2 | 64.3 | **0.456** | 66.9 | 59.2 | **0.373** |
| **Ki67 (median)** | 62.0 | 62.0 |  | 71.0 | 56.0 |  |
| **MDM2 > 5** | 35 | 27 | **0.518** | 20 | 10 | **0.315** |
| **MDM2 < 5** | 16 | 9 |  | 6 | 6 |  |
| **p16 1+2** | 9 | 6 | **0.905** | 5 | 1 | **0.243** |
| **p16 3** | 42 | 30 |  | 21 | 15 |  |
| **p27/Kip1 +** | 47 | 28 | **0.870** | 24 | 16 | **0.418** |
| **p27/Kip1 -** | 6 | 4 |  | 1 | 0 |  |
| **PD-L1 +** | 2 | 2 | **0.651** | 1 | 1 | **0.721** |
| **PD-L1 -** | 57 | 36 |  | 30 | 18 |  |
| **PTEN +** | 34 | 20 | **0.859** | 19 | 14 | **0.296** |
| **PTEN -** | 22 | 14 |  | 11 | 4 |  |
| **SMA 0+1** | 54 | 36 | **0.746** | 27 | 17 | **0.413** |
| **SMA 2+3** | 4 | 2 |  | 4 | 1 |  |
| **TILs +** | 21 | 12 | **0.458** | 13 | 7 | **0.571** |
| **TILs -** | 30 | 24 |  | 13 | 10 |  |
| **Vimentin +** | 32 | 19 | **0.543** | 21 | 9 | **0.137** |
| **Vimentin -** | 22 | 17 |  | 6 | 7 |  |

Age, age at diagnosis (years); BLBC CK, basal-like breast cancer (based on cytokeratins); TILs, tumor-infiltrating lymphocytes

*Student´s t-test
